# hERG activators exhibit antitumor effects in breast cancer through calcineurin and β-catenin-mediated signaling pathways

**DOI:** 10.3389/fphar.2025.1545300

**Published:** 2025-01-23

**Authors:** Yan Yu, Chengchun Zhu, Xiao Wang, Ying Shi, Yiping Gao, Zhiyi Yu

**Affiliations:** Department of Medicinal Chemistry, School of Pharmaceutical Sciences, Cheeloo College of Medicine, Shandong University, Jinan, Shandong, China

**Keywords:** hERG activator, antitumor mechanisms, triple negative breast cancer, ER+ breast cancer, combination therapy

## Abstract

**Background:**

Breast cancer remains a leading cause of mortality among women worldwide, with existing therapeutic options often accompanied by significant side effects and a persistent risk of disease recurrence. This highlights the need for novel drug candidates with new mechanisms of action by targeting alternative signaling pathways. While hERG channel is notoriously regarded as an off-target due to drug-induced cardiotoxicity, its therapeutic potential as a drug target remains largely unexplored.

**Methods:**

This study investigated the role of hERG in breast cancer progression and its impact on patient survival. The anti-proliferative, anti-migratory, anti-invasive and pro-apoptotic effects of hERG activators were evaluated using the Cell Counting Kit-8, wound healing assay, transwell assay and cell apoptosis assay, respectively. Western blotting, Ca^2+^ imaging and immunofluorescence assays were employed to study their antitumor mechanisms of actions.

**Results:**

We identified two novel hERG activators, **SDUY429** and **SDUY436**, which effectively inhibited the proliferation and migration of MDA-MB-231 and MCF-7 cells. In addition, **SDUY436** demonstrated significant anti-invasive and pro-apoptotic effects in MDA-MB-231 cells. Mechanistically, the anti-proliferative activity of hERG activators were mediated through calcineurin activation via enhanced calcium ion influx, which facilitated the nuclear translocation of nuclear factor of activated T cells (NFAT) and upregulated p21^Waf/Cip^ expression. Furthermore, both **SDUY429** and **SDUY436** remarkably suppressed the migration and invasion of MDA-MB-231 cells by downregulating the protein kinase B (AKT)/glycogen synthase kinase-3 beta (GSK3β)/β-catenin signaling pathway. The observed reduction in phospho-AKT-Ser473 (*p*AKT^S473^) expression resulted in the decreased levels of phospho-GSK3β-Ser9 (*p*GSK3β^S9^), thereby limiting the nuclear localization of β-catenin, which led to the inhibition of cell migration and invasion. Notably, combining **SDUY429** or **SDUY436** with the AKT inhibitor MK-2206 produced synergistic anti-proliferative effects.

**Conclusion:**

These findings suggest that hERG activators hold promise as new potential therapeutic agents for the treatment of breast cancer, paving the way for future investigations into their clinical applications.

## 1 Introduction

Breast cancer remains the most commonly diagnosed malignancy and the leading cause of cancer-related mortality among women worldwide (Siegel, et al., 2021; Sung, et al., 2021; Wilkinson and Gathani, 2022). Conventional treatment approaches for breast cancer consist of radiation therapy, hormone blockade therapy and chemotherapy. Despite the effectiveness of these treatments, 30%–50% of patients still suffer from metastatic recurrences that may occur months or even decades after the initial diagnosis (Chambers, et al., 2002; Riggio, et al., 2021; Weigelt, et al., 2005). Breast cancer is classified into subtypes based on immunohistochemical biomarkers, including estrogen receptor (ER), progesterone receptor (PR) and human epidermal growth factor receptor 2 (HER2) (Yeo and Guan, 2017). Clinically, these biomarkers not only define the disease but also guide prognosis and therapeutic decisions. Triple-negative breast cancer (TNBC), characterized by the absence of ER, PR and HER2 expression, accounts for 15%–20% of breast cancer cases (Harbeck, et al., 2019). Due to the lack of effective therapeutic targets, TNBC is known for its aggressive clinical behavior, high rate of early relapse and poor overall prognosis. Currently, chemotherapy remains the standard treatment for TNBC (Harbeck, et al., 2019). Regimens based on docetaxel and vinorelbine have shown significant efficacy in early and advanced TNBC, respectively (Caparica, et al., 2019). However, resistance to these agents is common, highlighting the necessity for more effective therapeutic strategies. In addition, approximately 70% of breast cancer are ER-positive (DeSantis, et al., 2017). Endocrine therapies targeting ER, such as selective ER modulators (e.g., tamoxifen), selective ER degraders (e.g., fulvestrant) and aromatase inhibitors (e.g., letrozole and anastrozole), have been effective in reducing recurrence for up to 5 years in early ER^+^ breast cancer (Bray, et al., 2018; DeSantis, et al., 2017; Williams and Harris, 2014). Nevertheless, hormonal resistance to these therapies often leads to metastatic progression (Weiss, et al., 2022). Collectively, there is an urgent need to identify drug candidates with new targets and mechanism of actions for the treatment of breast cancer.

The hERG (K_v_11.1) channel, a member of the voltage-gated K^+^ channel family encoded by the human *ether-à-go-go*-related gene, is predominantly distributed in cardiac tissues (Raschi, et al., 2008), while it is also present in neurons, neuroendocrine glands and smooth muscle. The hERG channel mediates the rapid delayed rectifier K^+^ current (*I*_*Kr*_) and plays a critical role in phase Ⅲ repolarization of the action potential duration (Sanguinetti, et al., 1995). A number of studies have unraveled that dysfunction of hERG channel through drug-induced blockade or loss-of-function mutations significantly reduces the *I*_*Kr*_, leading to acquired and congenital long QT syndromes (LQTSs), respectively (Curran, et al., 1995; Trudeau, et al., 1995). Due to the promiscuity of the channel’s lumen site, a variety of drugs with diverse chemical structures are associated with hERG-induced cardiotoxicity, including antiarrhythmics, psychoactive drugs, antibiotics and antimalarial drugs (Seebohm, 2005). Consequently, much attention has been devoted to understanding the hERG channel’s role in cardiac repolarization to circumvent the adverse effects of channel blockers. Beyond its cardiac functions, accumulating evidence implies that the hERG channel may be dysregulated in various cancers, potentially contributing to tumor cell proliferation (He, et al., 2020). For example, the hERG activator, NS1643, has shown promise in inhibiting the growth of breast cancer cells, making it a potential candidate for cancer therapy (Fukushiro-Lopes, et al., 2018; Senyuk, et al., 2021). Activation of the hERG channel by NS1643 is associated with accelerated cellular senescence (Lansu and Gentile, 2013) and ROS-dependent DNA damage in breast cancer cells (Fukushiro-Lopes, et al., 2018). Furthermore, hERG activators have demonstrated antitumor effects by activating NFAT/p21 (Perez-Neut, et al., 2016) and calpain/protein tyrosine phosphatases 1B/Caveolin-1 (Cav-1) signaling pathways (Jiang, et al., 2022), while simultaneously inhibiting the Wnt/β-catenin signaling cascade (Breuer, et al., 2019). Tumorigenesis often involves a disruption of the balance between cell proliferation and apoptosis, with the cyclin-dependent kinase inhibitor p21 serving as a crucial regulator (Abbas and Dutta, 2009; Harper, et al., 1993). The p21 protein inhibits the activity of cyclin-dependent kinase-cyclin complexes and proliferating cell nuclear antigen (Abbas and Dutta, 2009), resulting in the suppression of tumor cell growth. Additionally, activation of Wnt/β-catenin signaling can compromise cancer immune surveillance and reduce the efficacy of immunotherapies like immune checkpoint inhibitors (Du, et al., 2020; Galluzzi, et al., 2019). Cav-1, which is involved in caveolae formation and cell signaling regulation (Bhowmick, et al., 2023), has also been implicated in cell invasion, migration and metastasis in breast cancer (Martínez-Meza, et al., 2019). Interestingly, hERG blockers have been reported to inhibit the proliferation of tumor cells. For instance, cisapride, a prototypical hERG blocker, has been found to effectively arrest the growth of gastric cancer cells, highlighting the channel’s potential as a therapeutic target for cancer treatment (Shao, et al., 2005). Altogether, these findings suggest that the hERG channel represents a promising drug target for cancer therapy, with both activation and inhibition potentially influencing cancer progression depending on the cancer types. To date, only hERG activators have been identified as potential drug candidates for breast cancer. However, research on hERG activators in breast cancer remains limited, which impedes a comprehensive understanding of their therapeutic potential.

In this study, we demonstrate that novel hERG activators, **SDUY429** and **SDUY436**, significantly suppressed the proliferation of MDA-MB-231 breast cancer cells by activating the NFAT/p21 signaling pathway and inducing cell apoptosis. Moreover, these activators exerted the anti-migratory and anti-invasive properties in MDA-MB-231 cells by interrupting the AKT/GSK3β/β-catenin signaling cascade. When combined with the AKT inhibitor MK-2206, **SDUY429** and **SDUY436** exhibited profound synergistic anti-proliferative effects against MDA-MB-231 cells. These findings shed new light on the mechanisms by which hERG activators inhibit the proliferation, migration and invasion of breast cancer cells, offering new therapeutic opportunities to prevent breast cancer progression.

## 2 Materials and methods

### 2.1 Cells, antibodies and reagents

All cell lines were generously provided by Dr. Xiuli Guo from the School of Pharmaceutical Sciences at Shandong university. MDA-MB-231 (Wang, et al., 2022) and MCF-7 (Zhang, et al., 2014) cells were cultured in DMEM (Gibco, Cat. # 31600034, American), supplemented with 10% fetal bovine serum (FBS) (ExCell Bio, Cat. # FSP500, China) and 10 μL/mL penicillin/streptomycin (Solarbio, Cat. # P1400, China). The cells were maintained at 37°C in a humidified atmosphere containing 5% CO_2_. Rabbit anti-*p*AKT^S473^ (Cat. # 4060T, RRID: AB_2315049), AKT (Cat. # 4691T, RRID: AB_915783), p21^waf/cip^ (Cat. # 2947T, RRID: AB_823586) and NFAT (Cat. # 5861T, RRID: AB_10834808) antibodies were purchased from Cell Signaling Technologies (CST, American). Rabbit anti-*p*GSK3β^S9^ (Cat. # 11002, RRID: AB_895371) and GSK3β antibodies (Cat. # 21002, RRID: AB_895369) were purchased from Signalway Antibody (SAB, American). Rabbit anti-β-catenin (Cat. # CY3523, RRID: AB_3668890), Histone H_3_ (Cat. # CY6587, RRID: AB_2889879), β-actin (Cat. # AB0035, RRID: AB_2904142), GAPDH (Cat. # AB0037, RRID: AB_2891315) and Goat anti-rabbit IgG secondary antibodies (Cat. # AB0101, RRID: AB_2941855) were purchased from Abways (China). The alexa fluor 594 conjugated anti-rabbit secondary antibody (Cat. # bs-0295G-AF594, RRID: AB_2940825) and *p*GSK3β^Y216^ (Cat. # bs-4079R, RRID: AB_11070412) antibody were obtained from Bioss (China).

### 2.2 Effect of *KCHN2* expression on patient survival

The TISIDB database (http://cis.hku.hk/TISIDB/index.php) was used to analyze the expression of the *KCNH2* gene across various cancers. The association between *KCNH2* gene expression and survival rates of patients with breast cancer was explored. RNA-sequencing profiles and clinical data for breast cancer were obtained from the TCGA dataset (https://portal.gdc.com). For Kaplan-Meier curves, p-values and hazard ratios (HRs) with 95% confidence intervals (CIs) were generated by log-rank tests and univariate cox proportional hazards regression. All analyses were performed using R software (Foundation for Statistical Computing 2020, version 4.0.3). Patients with various subtypes of breast cancer, including TNBC and ER^+^ breast cancer, were selected as subjects for analysis to predict the overall survival. The Kaplan-Meier survival analysis was performed on the gene signature derived from the TCGA dataset, with statistical comparisons performed using the log-rank tests. The results include HR values, 95% CIs and the median survival times (LT50) for each group under analysis.

### 2.3 Cell viability assay

Cell viability was evaluated using the Cell Counting Kit-8 (CCK-8) assay. Cells in the logarithmic growth phase (8,000 cells/well) were seeded into 96-well plates. After overnight incubation to allow attachment, cells were treated with different concentrations of test compounds for 24 or 48 h. Following treatment, 10 μL of the CCK-8 reagent (Dojindo, Cat. # CK04, China) was added to each well. The cells were then incubated at 37°C in 5% CO_2_ for 45 min. The UV absorbance was measured at 450 nm to assess cell proliferation. The data were analyzed using GraphPad Prism 9.0.

### 2.4 Wound healing assay

When MDA-MB-231 or MCF-7 cells reached 90% confluence in 6-well plates, the wounds in each well were created using pipette tips after dividing the plate into different regions by drawing straight lines on the bottom. Detached cells were removed by washing with phosphate buffered solution (PBS) buffer (2.0 mM KH_2_PO_4_, 8.0 mM Na_2_HPO_4_·12H_2_O, 136.0 mM NaCl, 2.6 mM KCl). Cells were then incubated in medium containing 1% FBS and various concentrations of **SDUY429** or **SDUY436** for 24 h. Images were captured at 0 and 24 h using a microscope (Olympus, Japan), and the wound areas were quantified using ImageJ 1.45 software.

### 2.5 Transwell assay

Invasion assays were performed in 24-well plates (Nest) using chambers with 8 µm pore transparent PET filters (LABSELECT, China). Matrigel (Corning, Cat. # 356234, American) was diluted 1:9 (MDA-MB-231 cells) or 1:20 (MCF-7 cells) with serum-free medium, transferred to the transwell inserts and incubated at 37°C for 1 h to allow gelation. MDA-MB-231 cells (15,000 cells/well) or MCF-7 cells (20,000 cells/well) in 100 µL of serum-free DMEM medium were seeded into the upper chamber and incubated for 24 h with vehicle or desired concentrations of **SDUY429** or **SDUY436**. The lower chamber was filled with 500 µL of DMEM medium containing 10% FBS. After incubation, the matrigel-containing upper chamber was washed with PBS, fixed with 4% paraformaldehyde for 30 min and stained with 0.1% crystal violet for 30 min. Non-invading cells were removed with a cotton swab, and the chambers were rinsed with distilled water. The number of invading cells was visualized using a microscope (Olympus, Japan) and quantified via ImageJ 1.45 software.

### 2.6 Apoptosis analysis

When MDA-MB-231 cells reached 70% confluence in 6-well plates, they were treated with various concentrations of **SDUY429** and **SDUY436** for 24 h. Apoptosis was evaluated using the Cell Apoptosis Kit (APExBIO Cat. No. K2003) according to the manufacturer’s instructions. In short, 5 × 10^5^ cells were resuspended in 500 μL of binding buffer. The cell suspension was then gently mixed with 5 μL of Annexin V-FITC and 5 μL of propidium iodide (PI) working solution. The mixture was incubated for 15 min in the dark to allow for the binding of Annexin V-FITC to phosphatidylserine on the cell surface and the permeation of PI into compromised cell membranes. The stained cells were analyzed via flow cytometry. The fluorescence of Annexin V-FITC and PI was measured, allowing for the quantification of apoptotic cells. The data were used to categorize the cell populations into viable, early apoptotic, late apoptotic and necrotic cells based on their distinct fluorescence signatures.

### 2.7 Ca^2+^ imaging assay

Cells in the logarithmic growth phase (15,000 cells/well) were seeded into 96-well plates. The Ca^2+^ fluorescent probe (Fluo-4 AM, Beyotime, China) was diluted to 1 μM with PBS buffer containing 1 mM probenecid. When cells reached 90% confluence, they were incubated with the Ca^2+^ fluorescent probe for 20 min at 28°C. After discarding the probe solution, cells were washed with PBS buffer and imaged immediately using a KEYENCE BZ-X800E microscope (KEYENCE, Japan) following the treatment with various concentrations of **SDUY429** and **SDUY436**.

### 2.8 Immunofluorescence staining

MDA-MB-231 cells were seeded onto gelatin-coated glass coverslips. After 24-h treatment of **SDUY429** and **SDUY436** in basic DMEM medium, cells were washed with cold PBS buffer, fixed with 4% paraformaldehyde and permeabilized with 0.3% Triton-X 100 for 15 min. Cells were then blocked with 5% bovine serum albumin (BSA) for 1 h at room temperature, followed by overnight incubation with primary antibodies at 4°C. On the next day, cells were incubated with secondary antibodies for 1 h in the dark at room temperature, and DAPI was added for 5 min in the dark to stain nuclei. Images were captured using a microscope (Olympus, Japan).

### 2.9 Western blotting

When MDA-MB-231 cells reached 70% confluence in 6-well plates, they were treated with various concentrations of test compounds for 24 h. Cells were lysed using RIPA lysis buffer (Beyotime, China) supplemented with 1 × phosphatase inhibitors (Biosharp, China) and 1 μM phenylmethanesulfonyl fluoride (Biosharp, China). The lysates were centrifuged at 12,000 rpm at 4°C for 15 min, and the protein concentrations were determined using a BCA protein assay kit (Solarbio,Cat. # PC0020, China). Proteins were mixed with 5 × loading buffer (Solarbio, China) and heated to 95°C for 7 min. Equal amounts of proteins were resolved on 10%–12% sodium dodecyl sulfate-polyacrylamide electrophoresis gels and transferred onto 0.22 μM polyvinylidene fluoride membranes (Merck Millipore, Germany). After blocking with nonfat milk in TBST buffer (20 mM Tris, 150 mM NaCl, and 0.1% Tween 20, pH 7.6) at 37°C for 1 h, the membranes were incubated with primary antibodies (GAPDH, 1:10,000; AKT, 1:2000; *p*AKT^S473^, 1:2000; β-actin, 1:10,000; *p*GSK3β^S9^, 1:1,000; *p*GSK3β^Y216^, 1:1,000; GSK3β, 1:1,000; β-catenin, 1:1,000; Histone H_3_, 1:10,000; p21, 1:1,000) overnight at 4°C. On the next day, the membranes were incubated with HRP-conjugated secondary antibodies (Goat anti-Rabbit IgG (H+L), 1:10,000) for 1 h at room temperature. The protein bands were visualized using a chemiluminescence system, and the densitometric analysis was performed using ImageJ 1.45 software.

### 2.10 Co-administration experiment

When MDA-MB-231 cells reached 60% confluence in 96-well plates, they were treated with either hEGR activators alone (25 μM and 50 μM) or in combination with an AKT inhibitor (1 μM, 3 μM and 10 μM) for 48 h. Afterwards, 10 μL of the CCK-8 reagent (Dojindo, China) was added into each well. The cells were then incubated at 37°C in 5% CO_2_ for 45 min. The UV absorbance was measured at 450 nm to assess cell proliferation. The data were analyzed using GraphPad Prism 9.0.

Next, the interactions between hERG activators and the AKT inhibitor were characterized using CompuSyn software to determine the combination index (CI) (Chou, 2010). The effect of the drug combination was analyzed using the CI method defined by the following equation: CI = (Inhibition)_AB_/[(Inhibition)_A_ + (Inhibition)_B_], where (Inhibition)_AB_ represents the inhibition rate when hEGR activators and the AKT inhibitor are used in combination, whereas (Inhibition)_A_ and (Inhibition)_B_ represent the inhibition rates of groups treated with hERG activators and AKT inhibitors alone, respectively. CI > 1 indicates antagonism, CI = 1 indicates additivity, CI < 1 indicates synergy, and CI < 0.7 indicates significant synergy. Each CI value was calculated as the mean from at least three independent experiments.

$$CI=\frac{{\left(Inhibition\right)}_{AB}}{{\left(Inhibition\right)}_{A}+{\left(Inhibition\right)}_{B}}$$

For further investigation of the co-administration mechanism, MDA-MB-231 cells were cultured in 6-well plates to reach a 60% confluence. The cells were subjected to two different treatment regimens for 48 h: one group treated with hERG activators at 25 μM and the other group treated with a combination of hERG activators at 25 μM and the AKT inhibitor at 10 μM. Western blotting was conducted as previously described to evaluate the expression levels of proteins associated with the cellular responses to these treatments.

### 2.11 Data analysis and statistics

All statistical analyses were performed using GraphPad Prism 9.0 with statistical significance set at p ≤ 0.05. Data are presented as mean ± SEM. One-way ANOVA was used for multiple group comparisons, while Student’s t-test was employed for comparisons between two groups.

## 3 Results

### 3.1 hERG activators inhibited the proliferation, migration and invasion of breast cancer cells and exhibited pro-apoptotic effects

**FIGURE 1:**
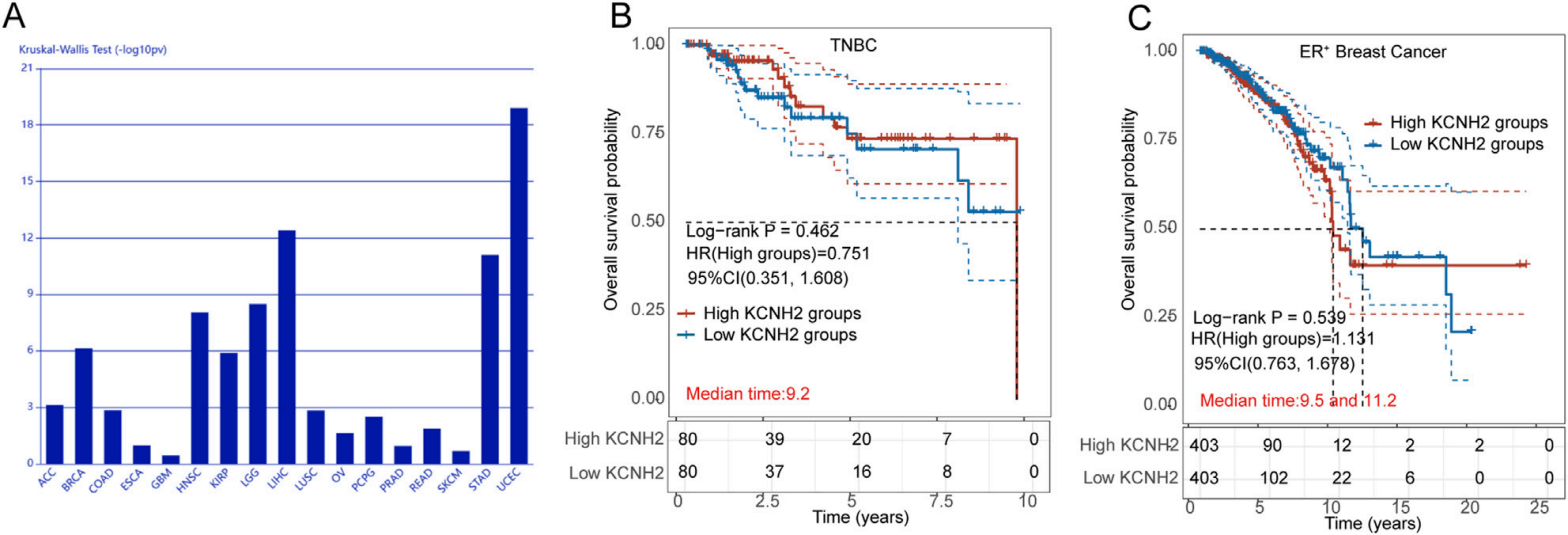
**(A)** Association between *KCNH2* expression and immune subtypes across human cancers; **(B)** Survival analysis of TNBC patients with high (red) and low (blue) expression of *KCNH2* gene; **(C)** Survival analysis of ER^+^ breast cancer patients with high (red) and low (blue) expression of *KCNH2* gene.

Initially, we analyzed the expression of the hERG gene (*KCNH2*) across an array of cancer types and observed a significant correlation with breast cancer (Figure 1A). To further explore the role of hERG channel in breast cancer prognosis, we utilized bioinformatic tools to investigate the survival rates of patients with high and low *KCNH2* expression. The HR for the high-expression group, compared to the low-expression group, served as an indicator. As displayed in Figure 1B, the HR for TNBC patients was 0.751, suggesting that TNBC patients with high *KCNH2* expression tended to have elevated survival rates. However, the correlation between *KCNH2* expression and the survival rates of ER^+^ patients was less pronounced with a HR of 1.131 (Figure 1C). These results suggest that hERG activators may exert weaker antitumor activity in ER^+^ cell lines compared to TNBC cell lines.

**FIGURE 2:**
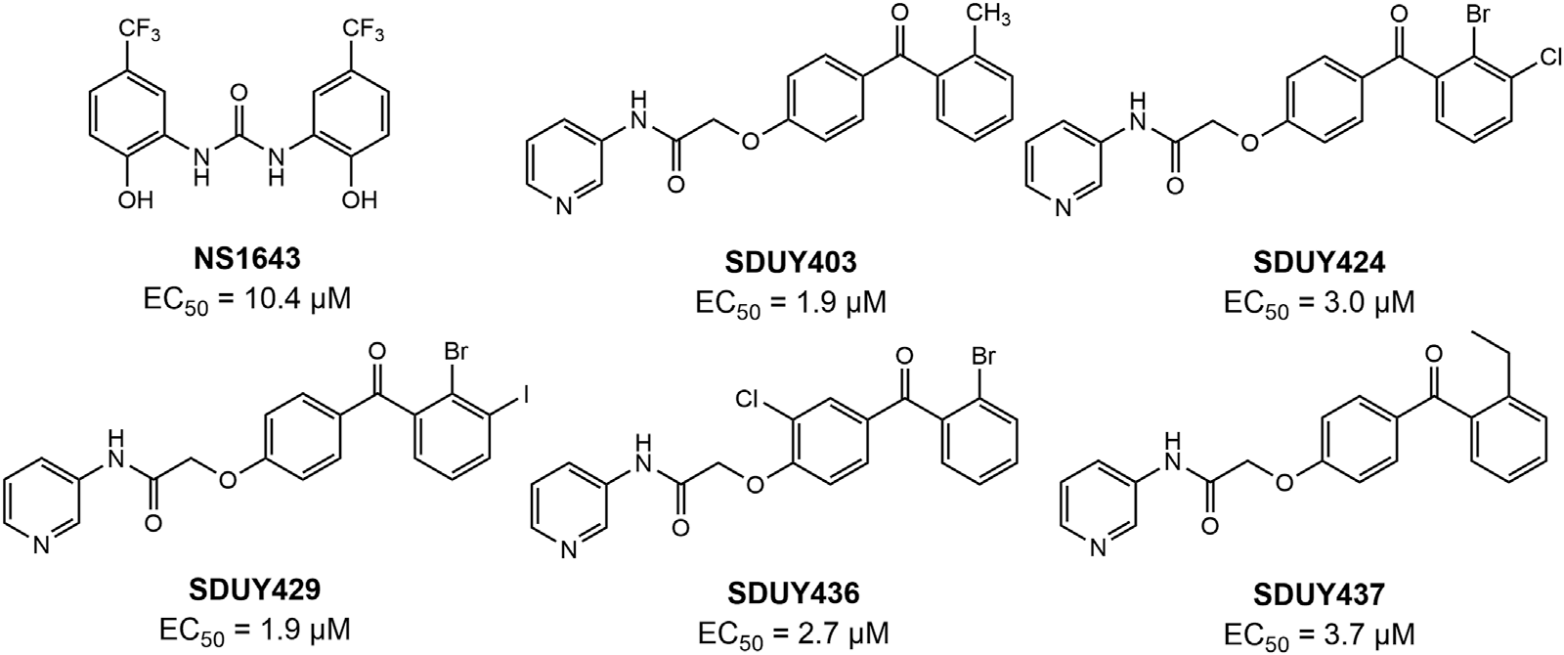
The chemical structures of NS1643 and new hERG channel activators.

**FIGURE 3:**
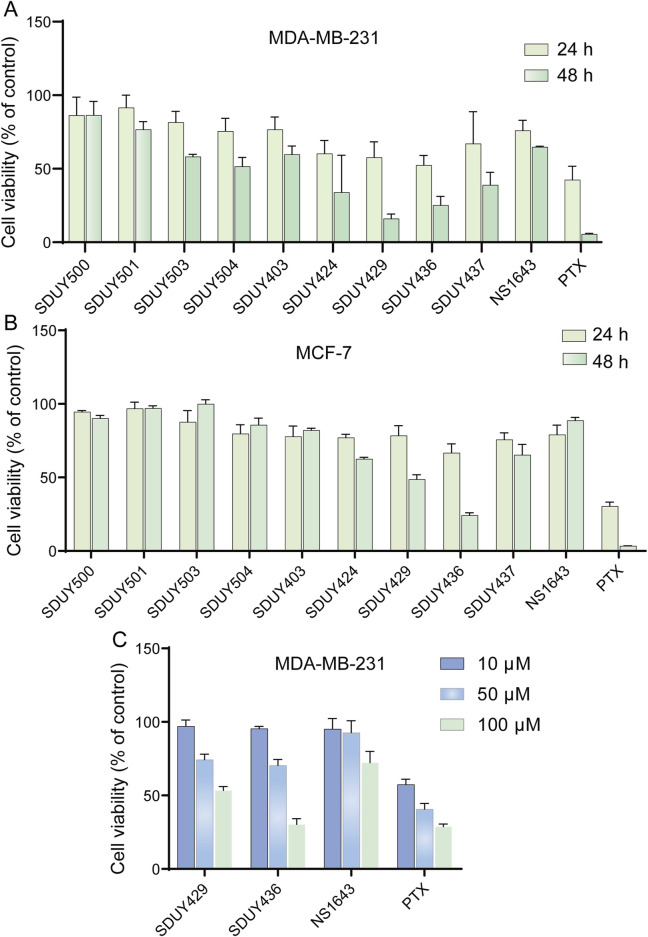
Assessment of breast cancer cell viability following hERG activator treatment. **(A)** The viability of MDA-MB-231 cells was determined using the CCK-8 assay following a 24- or 48-h exposure to 50 μM hERG activators or paclitaxel (PTX); **(B)** The viability of MCF-7 cells was determined using the CCK-8 assay following a 24- or 48-h exposure to 50 μM hERG activators or PTX; **(C)** The impacts of various concentrations (10, 50 and 100 μM) of **SDUY429**, **SDUY436**, NS1643 and PTX on MDA-MB-231 cell viability were evaluated using the CCK-8 assay after 24 h. All data are presented as mean ± SEM from three independent experiments.

In a previous study, we identified a series of hERG activators with new chemical structures, which enhanced K^+^ current and ameliorated LQTSs (Figure 2). Based on the bioinformatic analysis (Figure 1), we chose two breast cancer cell lines, including TNBC-derived MDA-MB-231 and ER^+^-derived MCF-7 cells, to explore the potential anti-proliferative effects of these hERG activators using the CCK-8 assay. As depicted in Figure 3A and Table 1, four hERG activators, **SDUY424**, **SDUY429**, **SDUY436** and **SDUY437**, markedly decreased the viability of MDA-MB-231 cells by more than 50% at a concentration of 50 μM after 48-h treatment. **SDUY429** and **SDUY436** were particularly effective, achieving 84.0% and 74.8% inhibition of cell viability, respectively. In contrast, these compounds displayed less pronounced anti-proliferative impacts on MCF-7 cell lines (Figure 3B), consistent with the survival analysis (Figures 1B, C). These findings indicate that activation of the hERG channel suppressed the proliferation of breast cancer cells with a more potent inhibitory effect in MDA-MB-231 cells compared to MCF-7 cells. Notably, **SDUY436** effectively inhibited the growth of MCF-7 cells with an inhibitory rate of 75.8% at 50 µM after 48 h. However, **SDUY429** showed reduced efficacy in MCF-7 cells with an inhibitory rate of 51.3%, suggesting cell-line-specific responses. Although both **SDUY429** and **SDUY436** was less potent than paclitaxel (PTX), their anti-proliferative activity surpassed that of NS1643 in both cell lines.

**TABLE 1:** Inhibition rates of hERG activators against MDA-MB-231 and MCF-7 cells at 50 μM after 24- and 48-h treatment periods.

Compounds	MDA-MB-231 cells		MCF-7 cells	
	Inhibition rates @ 50 μM (%, mean ± SEM)		Inhibition rates @ 50 μM (%, mean ± SEM)	
	24 h	48 h	24 h	48 h
SDUY500	13.6 ± 7.1	13.6 ± 5.4	5.5 ± 0.9	9.9 ± 2.0
SDUY501	8.4 ± 4.9	23.4 ± 3.1	3.2 ± 4.4	3.0 ± 1.7
SDUY503	8.4 ± 4.3	41.8 ± 0.9	12.4 ± 7.7	0.1 ± 2.9
SDUY504	24.4 ± 5.0	48.5 ± 3.6	20.4 ± 6.2	14.4 ± 4.7
SDUY403	23.3 ± 4.9	40.3 ± 3.2	22.2 ± 7.2	17.9 ± 1.4
SDUY424	39.7 ± 5.1	66.1 ± 14.6	23.0 ± 2.3	37.4 ± 1.1
SDUY429	42.3 ± 6.1	84.0 ± 1.8	21.6 ± 6.7	51.3 ± 3.0
SDUY436	47.6 ± 3.9	74.8 ± 3.4	33.3 ± 6.1	75.8 ± 1.7
SDUY437	32.9 ± 12.5	61.1 ± 5.0	24.3 ± 4.6	34.6 ± 7.1
NS1643	24.1 ± 4.1	35.2 ± 0.3	20.9 ± 6.5	11.4 ± 2.2
PTX	57.6 ± 5.3	94.4 ± 0.3	69.6 ± 2.7	96.6 ± 0.1

**SDUY429** and **SDUY436** were further assessed for their antitumor activity in MDA-MB-231 and MCF-7 cell lines through a panel of cellular experiments. As illustrated in Figure 3C, both **SDUY429** and **SDUY436** elicited a concentration-dependent reduction in cell viability of MDA-MB-231 cells within a 24-h exposure time. At 10 μM, neither **SDUY429**, **SDUY436**, nor NS1643 significantly inhibited cell growth, with the exception of PTX showing a 43.6% inhibition. At a concentration of 50 μM, **SDUY429** and **SDUY436** exhibited improved inhibitory activity of 25.7% and 29.6%, respectively, which surpassed the effect of NS1643 but remained less potent than PTX. Importantly, the inhibition rates for **SDUY436** and PTX (69.7% versus 71.2%) were almost equivalent at 100 μM, demonstrating the comparable efficacy of **SDUY436** to the established chemotherapeutical agent.

**FIGURE 4:**
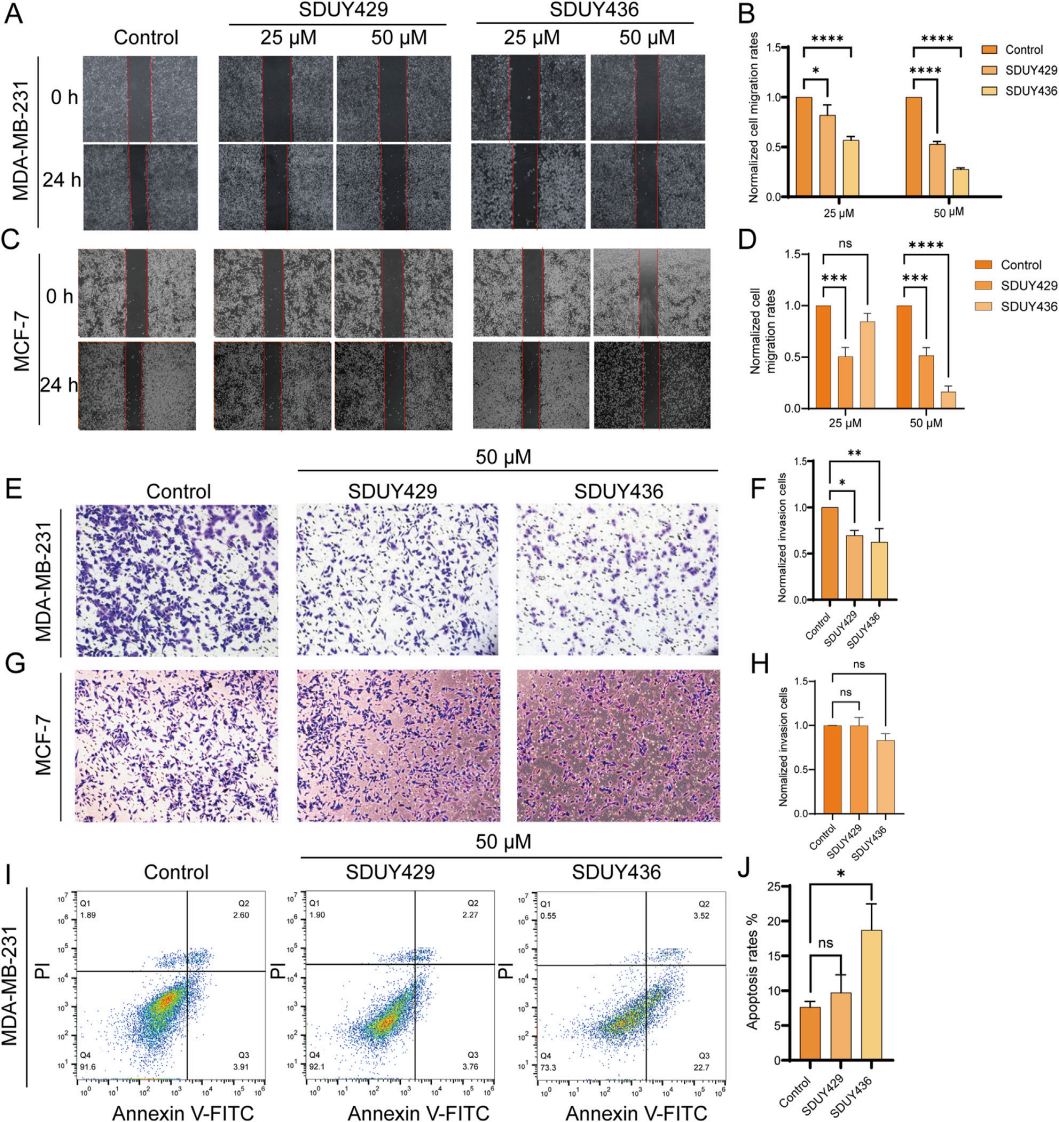
Impacts of hERG activators on the migration, invasion and apoptosis of breast cancer cells. **(A)** Wound healing experiments were conducted to assess the effects of varying concentrations (25 and 50 μM) of **SDUY429** and **SDUY436** on the motility of MDA-MB-231 cells with initial and final images captured at 0 and 24 h post-treatment; **(B)** Quantification of relative migration rates of MDA-MB-231 cells normalized to the control group following treatment with **SDUY429** and **SDUY436**; **(C)** Wound healing experiments were conducted to assess the effects of varying concentrations (25 and 50 μM) of **SDUY429** and **SDUY436** on the motility of MCF-7 cells with initial and final images captured at 0 and 24 h post-treatment; **(D)** Quantification of relative migration rates of MCF-7 cells normalized to the control group following treatment with **SDUY429** and **SDUY436**; **(E)** Transwell invasion assays were performed to evaluate the invasive capacity of MDA-MB-231 cells after a 24-h exposure to 50 µM **SDUY429** and **SDUY436**; **(F)** Quantification of the relative invasive cell numbers in MDA-MB-231 cells normalized to the control group; **(G)** Transwell invasion assays were performed to evaluate the invasive capacity of MCF-7 cells after a 24-h exposure to 50 µM **SDUY429** and **SDUY436**; **(H)** Quantification of the relative invasive cell numbers in MCF-7 cells normalized to the control group; **(I)** Apoptosis assay in MDA-MB-231 cells treated with 50 µM **SDUY429** or **SDUY436** for 24 h. The distributions of cell population in live (Q4), early apoptosis (Q3), late apoptosis (Q2) and dead cell subpopulations (Q1) are indicated; **(J)** Statistical data of apoptosis rates calculated as the sum of the early and late apoptotic cells. All data are presented as mean ± SEM from three independent experiments. Statistical significance is denoted as follows: *p < 0.05; **p < 0.01; ***p < 0.001, ****p < 0.0001 versus the control group, ns = not significant.

Furthermore, both **SDUY429** and **SDUY436** dose-dependently inhibited the migration and invasion of MDA-MB-231 cells in wound healing and transwell assays. As depicted in Figures 4A, B, **SDUY429** and **SDUY436** significantly reduced the cell migration with inhibitory rates of 47.2% and 72.4% at 50 μM, separately. Notably, **SDUY436** was more effective than **SDUY429** in suppressing cell migration at both 25 μM and 50 µM. In MCF-7 cells, **SDUY429** significantly decreased the cell migration with inhibitory rates of 49.4% and 48.5% at 25 or 50 μM, respectively, while this effect was not concentration-dependent (Figures 4C, D). In contrast, **SDUY436** exerted no anti-migratory effect at 25 µM but was more effective than **SDUY429** in suppressing cell migration at 50 µM. In the transwell assay, **SDUY429** and **SDUY436** remarkably inhibited the invasion of MDA-MB-231 cells with inhibitory effects of 69.6% and 62.5% at 50 μM, respectively (Figures 4E, F). However, neither compounds demonstrated anti-invasion activity in MCF-7 cells. (Figures 4G, H). Collectively, these results highlighted the potential of **SDUY429** and **SDUY436** as promising therapeutic agents for the treatment of metastatic breast cancer. A comparative analysis of their activity against cell migration and invasion revealed similar efficacies between the two hERG activators in MDA-MB-231 cells.

Additonally, Annexin V/PI staining was employed to investigate the pro-apoptotic capabilities of **SDUY429** and **SDUY436**. As displayed in Figures 4I, J, **SDUY436** increased the apoptosis rate of MDA-MB-231 cells by 12% at 50 µM compared to the control group, whereas **SDUY429** did not show a significant pro-apoptotic effect.

### 3.2 **SDUY429** and **SDUY436** inhibited cell proliferation by increasing p21 expression

**FIGURE 5:**
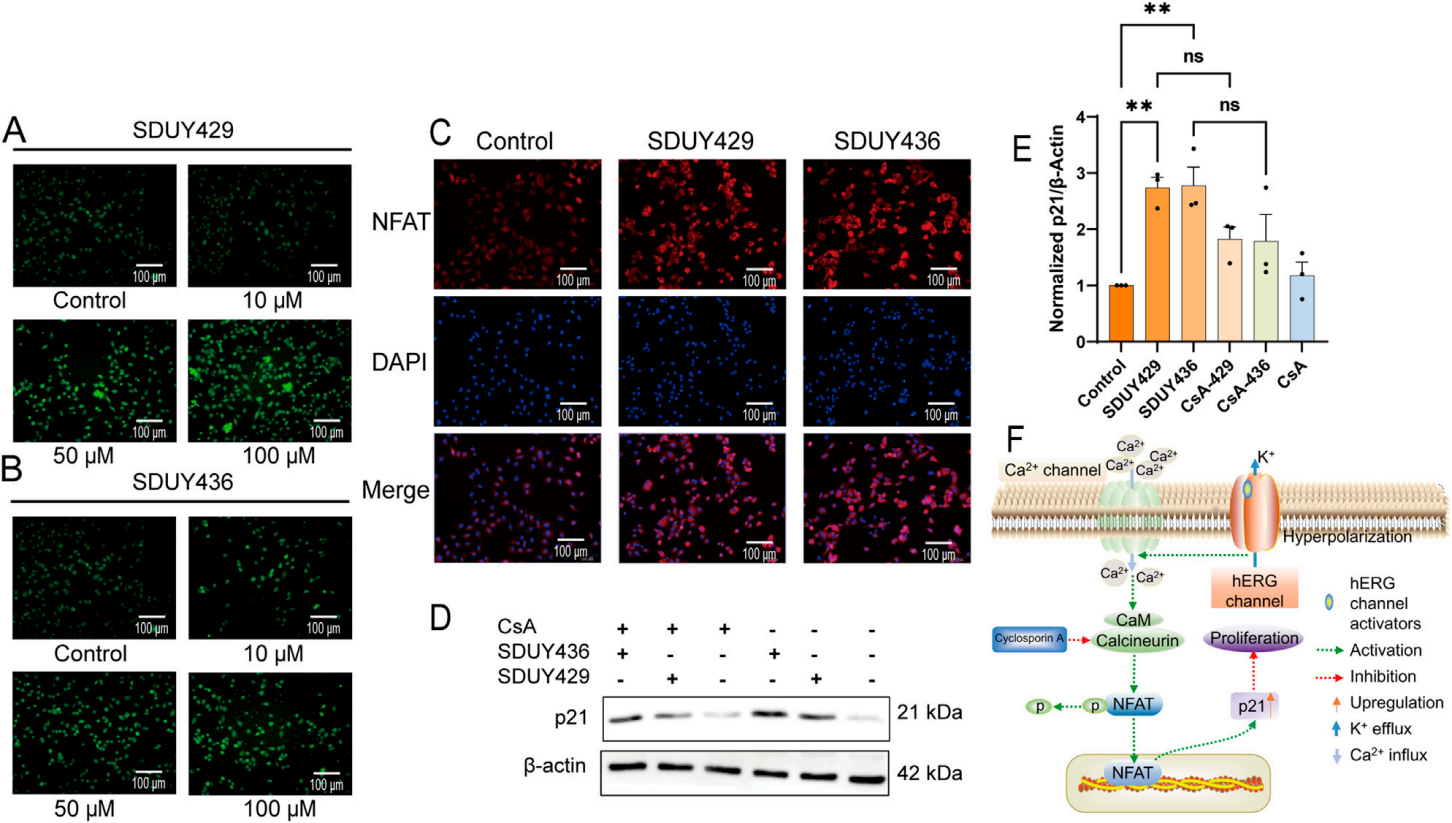
Mechanistic insights into the anti-proliferative effects of SDUY429 and SDUY436 on MDA-MB-231 cells. **(A)** Intracellular Ca^2+^ levels were measured in terms of relative fluorescence using the Fluo-4 AM probe, revealing a concentration-dependent increase in calcium levels upon treatment with **SDUY429**. Scale bars, 100 μm; **(B)** Intracellular Ca^2+^ levels were measured in terms of relative fluorescence using the Fluo-4 AM probe, revealing a concentration-dependent increase in calcium levels upon treatment with **SDUY436**. Scale bars, 100 μm; **(C)** Quantitative assessment of NFAT nuclear expression in MDA-MB-231 cells via immunofluorescence staining after a 24-h treatment with 50 μM **SDUY429** or **SDUY436**. Scale bar, 100 μm; **(D)** Western blotting analysis of p21 protein expression under various conditions: treatment with 10 μM CsA, 50 μM **SDUY429** or **SDUY436** alone, and a combination of 10 μM CsA with 50 μM **SDUY429** or **SDUY436**; **(E)** Quantification of relative p21 protein expression normalized to the control group; **(F)** Schematic representation of the anti-proliferative signaling pathways involving the hERG channel activation and the NFAT/p21 axis. All data are presented as mean ± SEM from at least three independent experiments. Statistical significance is indicated as follows: *p < 0.05; **p < 0.01; ***p < 0.001, ****p < 0.0001 versus the control group, ns = not significant.

In the calcium imaging assay, both **SDUY429** and **SDUY436** increased the intracellular calcium levels in a concentration-dependent manner (Figures 5A, B). Immunofluorescence studies further confirmed that both hERG activators obviously facilitated nuclear translocation of the NFAT (Figure 5C). Western blotting analysis revealed a notable upregulation of p21 protein expression in MDA-MB-231 cells treated with 50 µM **SDUY429** and **SDUY436** (Figure 5D). As illustrated in Figure 5E, **SDUY429** triggered an approximate 3-fold increase in p21 expression, which was comparable to the effect of **SDUY436**. Notably, the addition of Cyclosporin A (CsA), a calcineurin inhibitor, attenuated the enhanced p21 expression by hERG activators, indicating the involvement of calcineurin in NFAT activation and the subsequent increase in p21 expression. Collectively, the hERG activators were able to induce the membrane hyperpolarization and augment calcium influx, thereby promoting calcineurin-induced NFAT nuclear translocation and elevated p21 expression (Figure 5F). These results highlight the potential of hERG activators in disrupting intracellular signaling pathways associated with cell proliferation and differentiation.

### 3.3 **SDUY429** and **SDUY436** suppressed cell motility by regulating nuclear β-catenin expression

**FIGURE 6:**
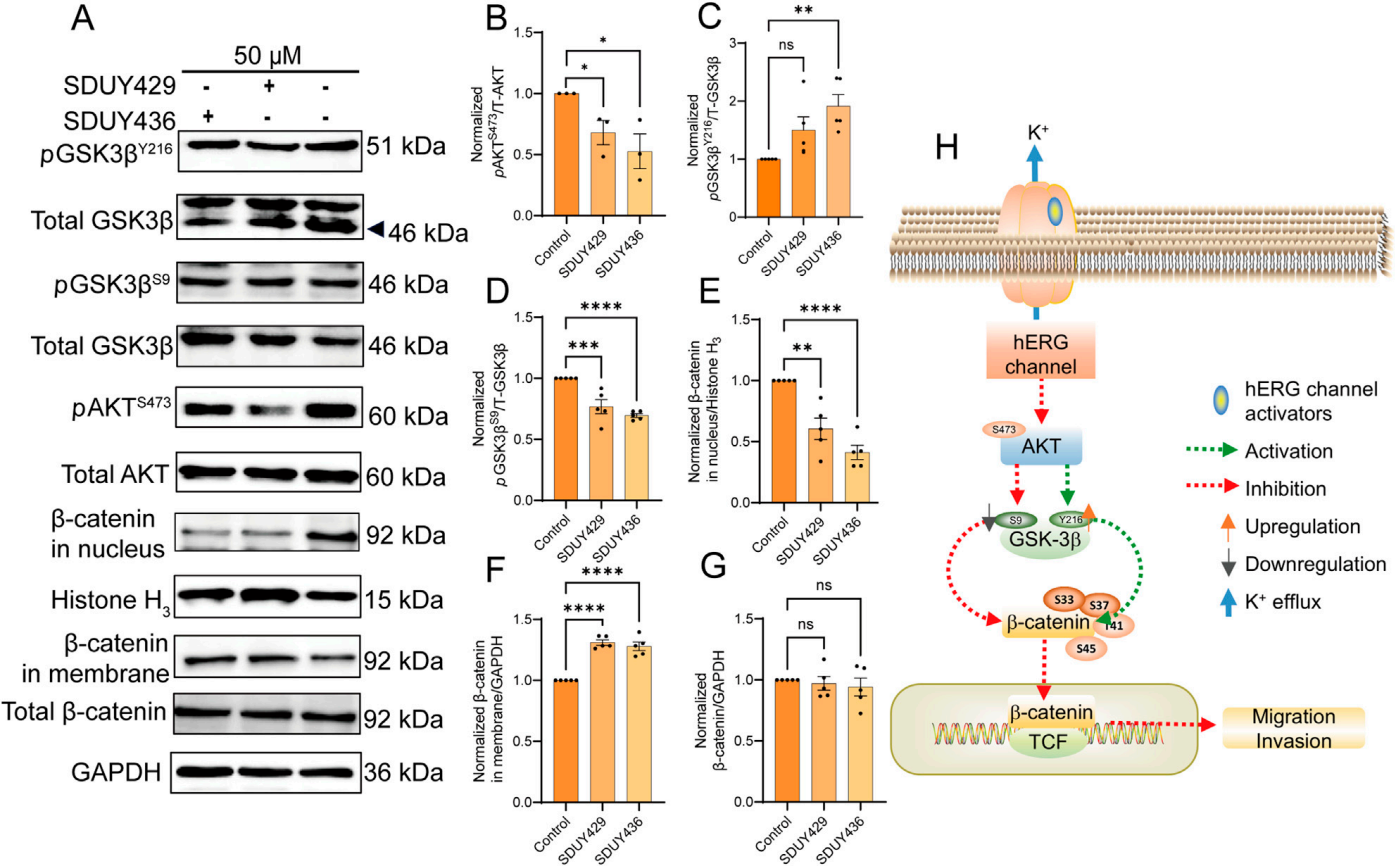
Mechanistic insights into the anti-migratory effects of SDUY429 and SDUY436 on MDA-MB-231 cells. **(A)** The expression levels of the *p*GSK3β^S9^, *p*GSK3β^Y216^, *p*AKT^S473^ and β-catenin proteins were evaluated by Western blotting after treatment with 50 μM **SDUY429** or **SDUY436** for 24 h; **(B)** Quantification of relative *p*AKT^S473^ protein expression normalized to the control group; **(C)** Quantification of the relative *p*GSK3β^Y216^ protein expression normalized to the control group; **(D)** Quantification of the relative *p*GSK3β^S9^ protein expression normalized to the control group; **(E)** Quantification of the relative β-catenin nuclear expression normalized to the control group; **(F)** Quantification of the relative β-catenin membrane expression normalized to the control group; **(G)** Quantification of the total β-catenin protein expression normalized to the control group; **(H)** Schematic representation of the anti-migratory signaling pathways involving the hERG channel activation and the AKT/GSK3β/β-catenin axis. All data are presented as mean ± SEM from at least three independent experiments. Statistical significance is denoted as follows: *p < 0.05; **p < 0.01; ***p < 0.001, ****p < 0.0001 versus the control group, ns = not significant.

Given the potent inhibitory effects of **SDUY429** and **SDUY436** on the migration and invasion of MDA-MB-231 cells (Figure 4), we further investigated the underlying mechanisms at the molecular level. As illustrated in Figures 6A, B, the expression levels of *p*AKT^S473^ were significantly reduced by **SDUY429** and **SDUY436**. Recognizing GSK3β as a crucial target of AKT, we evaluated the expression levels of GSK3β and found that the two hERG activators modulated its phosphorylation status. Specifically, these compounds enhanced the expression of active GSK3β (*p*GSK3β^Y216^) (Figure 6C) while diminished the expression of inactive GSK3β (*p*GSK3β^S9^) (Figure 6D). The reduction in *p*GSK3β^S9^expression prevented β-catenin translocation from the membrane to the nucleus. Consequently, we examined the influence of **SDUY429** and **SDUY436** on the expression and subcellular localization of β-catenin, a key regulator in the Wnt/β-catenin signaling pathway. Typically, Wnt/β-catenin signaling is activated within the cell through the redistribution and nuclear accumulation of β-catenin (Zhang, et al., 2011). Intriguingly, **SDUY429** and **SDUY436** impeded the nuclear translocation of β-catenin (Figure 6E) and enhanced its membrane localization (Figure 6F) without altering the total expression levels of β-catenin (Figure 6G). Overall, hERG activators inhibited the migration and invasion of breast cancer cells by suppressing the AKT/GSK3β signaling pathway and preventing the nuclear translocation of β-catenin (Figure 6H).

### 3.4 Combination of hERG activators with an AKT inhibitor enhanced the anti-proliferative effects

**FIGURE 7:**
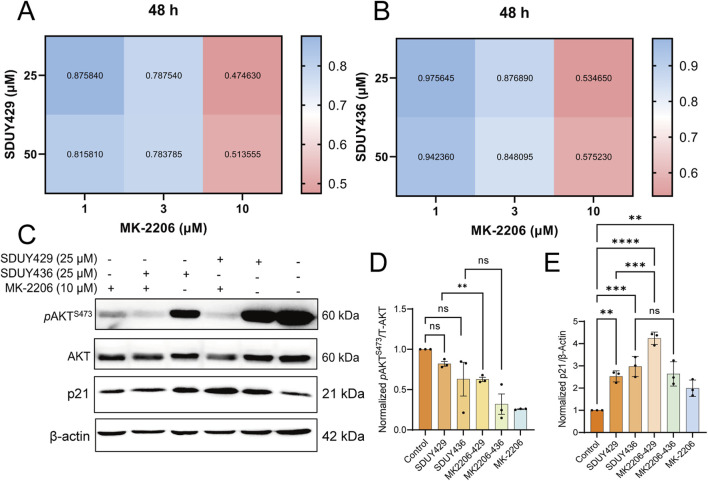
Synergistic effects of MK-2206 on the anti-proliferative activity of SDUY429 and SDUY436 in MDA-MB-231 cells. **(A)** A matrix of drug combinations was utilized to assess the synergistic effects between **SDUY429** and MK-2206 on MDA-MB-231 cell viability after 48 h, with CI values indicating the level of synergy; **(B)** A matrix of drug combinations was utilized to assess the synergistic effects between **SDUY436** and MK-2206 on MDA-MB-231 cell viability after 48 h with CI values indicating the level of synergy; **(C)** The expression levels of the *p*AKT^S473^ and p21 proteins were evaluated by Western blotting under various conditions: treatment with 10 μM MK-2206, 25 μM **SDUY429** or **SDUY436** alone, and a combination of 10 μM MK-2206 with 25 μM **SDUY429** or **SDUY436** for 48 h; **(D)** Quantification of the relative *p*AKT^S473^ protein expression normalized to the control group; **(E)** Quantification of the relative p21 protein expression normalized to the control group. All data are presented as mean ± SEM from at least three independent experiments. Statistical significance is indicated as follows: *p < 0.05; **p < 0.01; ***p < 0.001, ****p < 0.0001 versus the control group, ns = not significant.

Considering that p21 expression is typically downregulated by elevated levels of phosphorylated AKT, we postulated that a combinatorial therapeutic approach involving hERG activators and an AKT inhibitor MK-2206 might yield synergistic antitumor effects against breast cancer. To test this hypothesis, we evaluated the viability of MDA-MB-231 cells following a 48-h exposure to varying concentrations of **SDUY429** or **SDUY436** in combination with MK-2206 (Figures 7A, B). The CI analysis confirmed significant synergistic interactions, with CI values consistently below 0.55, particularly when MK-2206 at 10 µM was co-administrated with either **SDUY429** or **SDUY436** at 25 µM. Western blotting analysis further disclosed that the co-administration of **SDUY429** and MK-2206 markedly decreased *p*AKT^S473^ levels (Figures 7C, D) while significantly increased p21 expression relative to **SDUY429** alone (Figures 7C, E). Conversely, the combination of **SDUY436** with MK-2206 did not significantly alter the expression of *p*AKT^S473^ protein or enhance the p21 expression. This disparity in responses between **SDUY436** and **SDUY429** in conjunction with MK-2206 underscored the importance of compound selection in designing combination therapy. The favorable results observed with the **SDUY429** and MK-2206 combination suggest a promising therapeutic strategy for breast cancer. This approach leverages the dual effects of AKT inhibition and p21 upregulation to synergistically impede tumor progression.

## 4 Discussion

hERG is expressed in numerous human cancer cell lines and tissues but is absent in corresponding healthy cells (Cherubini, et al., 2000; Lastraioli, et al., 2004; Pillozzi, et al., 2002), suggesting that it may confer a selective advantage to tumor cells. Previous studies have highlighted the prevalence of the hERG channel in various breast cancer cell types, while it remains undetectable in normal breast cells (He, et al., 2020). The antitumor potential of the hERG activator NS1643 in breast cancer cells has been well- documented in a series of investigations (Breuer, et al., 2019; Fukushiro-Lopes, et al., 2018; Jiang, et al., 2022; Lansu and Gentile, 2013; Perez-Neut, et al., 2016). In this study, we extend this understanding by validating the antitumor activity of our in-house made hERG activators in breast cancer cells and further exploring the mechanisms underlying their anti-proliferative, anti-migratory and anti-invasive effects.

Our bioinformatic analysis uncovered a notable correlation between hERG gene expression and overall survival outcomes in patients with TNBC and ER^+^ breast cancer (Figure 1). Intriguingly, TNBC patients with elevated hERG gene expression exhibited improved overall survival rates compared to those with lower expression. Conversely, in ER^+^ patients, higher expression of the hERG gene was associated with poorer overall survival rates. The anti-proliferative studies further demonstrated that activation of the hERG channel effectively impeded the growth of MDA-MB-231 cells from the TNBC cell line, whereas its inhibitory potency was significantly reduced in MCF-7 cells from the ER^+^ breast cancer cell line, indicating cell-line-specific difference in responses. Collectively, these findings suggest that the hERG gene may serve as a protective factor in TNBC patients but has a more complex role in ER^+^ breast cancer.

Our experimental results showed that **SDUY429** and **SDUY436** significantly reduced the viability of MDA-MB-231 cells in a concentration-dependent manner and inhibited the cell migration and invasion. Both hERG activators exhibited stronger antitumor effect in MDA-MB-231 cells compared to MCF-7 cells. Moreover, **SDUY436** showed the pro-apoptotic effect against MDA-MB-231 cells (Figures 3, 4). Further investigation into the mechanisms of their anti-proliferative effects suggested that, akin to NS1643 (Perez-Neut, et al., 2016), these compounds might exert the antitumor activity via the NFAT/p21 signaling pathway in breast cancer. Activation of the hERG channel typically leads to potassium efflux and subsequent membrane hyperpolarization, which in turn facilitates the influx of calcium ions. The calcium imaging assay confirmed that **SDUY429** and **SDUY436** markedly enhanced calcium influx in MDA-MB-231 cells, triggering the activation of calcium-dependent enzymes that may ultimately result in cellular damage. Calcineurin, a Ca^2+^/calmodulin-dependent phosphatase, is known to activate NFATc1 in response to calcium signaling (Ivashkiv, 2009). Calcineurin promotes NFAT nuclear translocation by dephosphorylating NFAT. Consistent with these findings, our results from the immunofluorescence staining assay and Western blotting analysis indicated that the activation of calcineurin by increased calcium influx upon stimulation with **SDUY429** or **SDUY436** in MDA-MB-231 cells drove NFAT into the nucleus, contributing to elevated p21 expression. The p21 protein, recognized for its dual role in cancer development (Georgakilas, et al., 2017), acts as both a tumor suppressor and promoter depending on the cancer types. Studies have revealed that the delivery of a p21-p27 fusion gene into the breast cancer cell line suppresses its proliferation and induces the cell apoptosis (Jiang, et al., 2014; Shamloo and Usluer, 2019). Our findings elucidated the anti-proliferative mechanisms of **SDUY429** and **SDUY436** in MDA-MB-231 cells. These compounds initiated the activation of calcineurin via increased calcium influx, inducing the translocation of NFAT into the nucleus. The subsequent increase in nuclear localization of NFAT enhanced expression of the p21 protein, thereby contributing to the anti-proliferative effects of hERG activators. Although both **SDUY429** and **SDUY436** exerted profound anti-proliferative effects, only **SDUY436** displayed significant pro-apoptotic activity. This suggests that hERG activators may elicit distinct cellular responses across different breast cancer cell lines, with **SDUY436** exhibiting a more extensive inhibitory effect on MDA-MB-231 cells compared to **SDUY429**. The pro-apoptotic mechanism of hERG activators in breast cancer has not been previously characterized, and hence, we intend to explore this mechanism in future research endeavors.

A series of studies have unraveled that hERG activators suppress the migration and invasion of breast cancer cells by inhibiting the Wnt/β-catenin signaling pathway (Breuer, et al., 2019; Jiang, et al., 2022). In this study, we investigated the impacts of **SDUY429** and **SDUY436** on this signaling cascade. GSK3β and β-catenin are pivotal components of the Wnt/β-catenin pathway, and the phosphorylation status of GSK3β at Ser9 and Tyr216 is crucial for the regulation of β-catenin levels. *p*GSK3β^S9^, which inhibits GSK3β activity, leads to the accumulation of β-catenin in the nucleus, whereas *p*GSK3β^Y216^ that enhances GSK3β activity results in the suppression of β-catenin in nucleus (Li, et al., 2018). An increase in nuclear expression of β-catenin activates the Wnt/β-catenin pathway. Our results indicated that both **SDUY429** and **SDUY436** decreased the expression of *p*GSK3β^S9^ while enhanced the expression of *p*GSK3β^Y216^. Concurrently, these activators diminished the nuclear translocation of β-catenin. Altogether, these findings suggested that **SDUY429** and **SDUY436** exerted anti-migratory and anti-invasive effects in breast cancer by downregulating the Wnt/β-catenin signaling pathway. In addition, GSK3β is a known phosphorylation target of AKT (Moroi and Watson, 2015). AKT activation promotes the phosphorylation of GSK3β at Ser9, thereby inhibiting the degradation of β-catenin and activating the Wnt/β-catenin signaling pathway (Li, et al., 2014). Our data showed that both **SDUY429** and **SDUY436** reduced the expression of *p*AKT^S473^, implying their anti-migratory and anti-invasive efficacy against breast cancer by downregulating the AKT/GSK3β/β-catenin axis.

Numerous studies have confirmed that upregulating the p21 expression and inhibiting the PI3K/AKT signaling pathway effectively suppress cancer cell proliferation (Daaboul, et al., 2018; Ling, et al., 2018; Tian, et al., 2020). In this study, **SDUY429** and **SDUY436** enhanced the expression levels of p21 protein, thereby reducing the viability of MDA-MB-231 cells by more than 70% at 50 μM after a 48-h treatment period (Figure 3A). Additionally, treatment with 50 μM of **SDUY429** and **SDUY436** modestly reduced the expression of *p*AKT^S473^ protein (Figure 6A). These results are consistent with the established notion that the expression levels of phosphorylated AKT are inversely correlated with those of the p21 protein (Maheshwari, et al., 2022). Based upon these observations, we postulate that a combinatorial approach with an AKT inhibitor MK-2206 may potentiate the anti-proliferative effects of hERG activators in breast cancer. This hypothesis is supported by CI analysis, which demonstrated significant synergistic effects when **SDUY429** or **SDUY436** at 25 μM was co-administered with 10 μM of MK-2206, yielding CI values below 0.55. Western blotting analysis further confirmed the substantial upregulation of p21 protein expression and the downregulation of *p*AKT^S473^ levels with this combination, suggesting that the combination of hERG activators with AKT inhibitors holds promise as a promising therapeutic strategy in the treatment of breast cancer. Our findings indicated that the hERG channel could be a promising therapeutic target for breast cancer, with channel activators potentially serving as new antitumor therapeutics.

Due to the notorious cardiotoxicity associated with hERG blockade, researchers have been cautious about targeting this channel in drug discovery endeavors. Recently, the emergence of hERG activators presents a compelling opportunity to mitigate the congenital or drug-induced cardiotoxicity arising from hERG dysfunction. In this study, we have identified an additional and unexpected therapeutic potential for hERG activators as potential antitumor therapeutics in the treatment of breast cancer. We have thoroughly explored the intricate mechanisms underlying the anti-proliferative, anti-migratory and anti-invasive effects of two new in-house hERG activators, **SDUY429** and **SDUY436**, in breast cancer cells. While both compounds have demonstrated significant *in vitro* antitumor activity against TNBC and ER^+^ breast cancer, their efficacy may be insufficient to warrant immediate progression to *in vivo* pharmacodynamic and pharmacokinetic studies. Moreover, the antitumor mechanisms of actions for **SDUY429** and **SDUY436** require further validation through *in vivo* studies to support their potential for clinical applications. Additionally, the potential of hERG activators to shorten QT intervals and increase the risk of arrhythmias presents a notable safety concern that need to be addressed in future endeavors. Despite these limitations, our findings lay a critical foundation for the continued optimization and translational development of hERG activators as therapeutic agents for breast cancer.

## 5 Conclusion

In conclusion, the novel hERG activators, **SDUY429** and **SDUY436**, have demonstrated significant inhibitory efficacy on the proliferation, migration and invasion of breast cancer cells, including TNBC-derived MDA-MB-231 and ER^+^-derived MCF-7 cells. Notably, **SDUY436** also exhibits pro-apoptotic effects in MDA-MB-231 cells. Our comprehensive mechanistic investigations have revealed that these activators exert their antitumor activity by upregulating the NFAT/p21 pathway while downregulating AKT/GSK3β/β-catenin signaling cascade. Furthermore, combination therapy with hERG activators and AKT inhibitors elicits synergetic antitumor activity and provides additional confirmation of the underlying mechanisms of action. This study not only elucidates the connection between hERG activation and oncogenic processes but also paves the way for innovative clinical strategies in the diagnosis and treatment of breast cancer.

## Data Availability

The datasets presented in this study can be found in online repositories. The names of the repository/repositories and accession number(s) can be found in the article/Supplementary Material.

## Abbreviations

AKT, protein kinase B; BSA, bovine serum albumin; Cav-1, Caveolin-1; CCK-8, cell counting kit-8; CI, combination index; CsA, Cyclosporin A; ER, estrogen receptor; FBS, fetal bovine serum; GSK3β, glycogen synthase kinase-3 beta; hERG, human *ether-à-go-go*-related gene; HER2, human epidermal growth factor receptor 2; HR, hazard ratio; LQTSs, long QT syndromes; LT50, survival time; NFAT, nuclear factor of activated T cells; PBS, phosphate buffered solution; PI, propidium iodide; PR, progesterone receptor; *p*AKT^S473^, phospho-AKT-Ser473; *p*GSK3β^S9^, phospho-GSK3β-Ser9; *p*GSK3β^Y216^, phospho-GSK3β-Tyr216; ROS-dependent, reactive oxygen species-dependent; SEM, standard error of the mean; TNBC, triple-negative breast cancer.
