# Supplementary material for: hERG activators exhibit antitumor effects in breast cancer through calcineurin and β-catenin-mediated signaling pathways
Source: Front Pharmacol. 2025 Jan 23;16:1545300. doi: 10.3389/fphar.2025.1545300 (PMC11799564; doi:10.3389/fphar.2025.1545300)

**Supporting Information**

**hERG Activators Exhibit Antitumor Effects in Breast Cancer through Calcineurin and β-Catenin-mediated Signaling Pathways**

Yan Yu^a^, Chengchun Zhu^a^, Xiao Wang^a^, Ying Shi^a^, Yiping Gao^a^, Zhiyi Yu^a,*^

^a^Department of Medicinal Chemistry, School of Pharmaceutical Sciences, Cheeloo College of Medicine, Shandong University, Jinan, Shandong 250012, China

**^*^Corresponding authors:**

Zhiyi Yu Ph.D., Professor

School of Pharmaceutical Sciences, Cheeloo College of Medicine, Shandong University, China.

Phone: +86 13605416337

Email: zhiyi_yu@sdu.edu.cn

**Table of Contents**

1. **The structural characterization for SDUY429 and SDUY436**

2. ^1^H-NMR and ^13^C-NMR spectrum of **SDUY429 and SDUY436**

**The structural characterization for SDUY429 and SDUY436**

*2-(4-(2-bromo-3-iodobenzoyl) phenoxy)-N-(pyridin-3-yl) acetamide (****SDUY429****).* Light yellow power; m.p.142-143 ℃. ^1^H NMR (400 MHz, DMSO-*d*_6_) δ 10.40 (s, 1H), 8.78 (d, *J* = 2.6 Hz, 1H), 8.31 (dd, *J* = 4.7, 1.5 Hz, 1H), 8.11 (dd, *J* = 7.8, 1.5 Hz, 1H), 8.06 (ddd, *J* = 8.3, 2.6, 1.5 Hz, 1H), 7.75 - 7.69 (m, 2H), 7.42 (dd, *J* = 7.5, 1.5 Hz, 1H), 7.38 (dd, *J* = 8.3, 4.7 Hz, 1H), 7.28 (t, *J* = 7.7 Hz, 1H), 7.19 - 7.11 (m, 2H), 4.89 (s, 2H); ^13^C NMR (151 MHz, DMSO-*d*_6_) δ 193.56, 166.94, 162.99, 145.22, 142.90, 141.88, 141.52, 135.44, 132.65, 129.76, 128.73, 128.17, 127.31, 125.57, 124.10, 115.66, 104.77, 67.48. HR-MS m/z: calcd for C_20_H_14_BrIN_2_O_3_ [(M-H)^-^], 534.9154; found, 534.9106.

*2-(4-(2-bromobenzoyl)-2-chlorophenoxy)-N-(pyridin-3-yl) acetamide (****SDUY436****).* Light yellow power; m.p.120-121 ℃. ^1^H NMR (400 MHz, DMSO-*d*_6_) δ 10.49 (s, 1H, NH), 8.75 (d, *J* = 2.6 Hz, 1H), 8.30 (dd, *J* = 4.7, 1.4 Hz, 1H), 8.03 (dt, *J* = 8.3, 2.0 Hz, 1H), 7.84 - 7.68 (m, 2H), 7.62 - 7.44 (m, 4H), 7.37 (dd, *J* = 8.3, 4.7 Hz, 1H), 7.25 (d, *J* = 8.7 Hz, 1H), 5.05 (s, 2H); ^13^C NMR (151 MHz, DMSO-*d*_6_) δ 193.22, 166.36, 158.40, 145.19, 141.55, 140.31, 135.48, 133.37, 132.25, 131.41, 131.38, 130.04, 129.27, 128.35, 126.98, 124.16, 122.62, 118.80, 114.42, 67.99. HR-MS m/z: calcd for C_20_H_14_BrClN_2_O_3_ [(M-H)^-^], 444.9778; found, 444.9731.

The ^1^H NMR of **SDUY429**


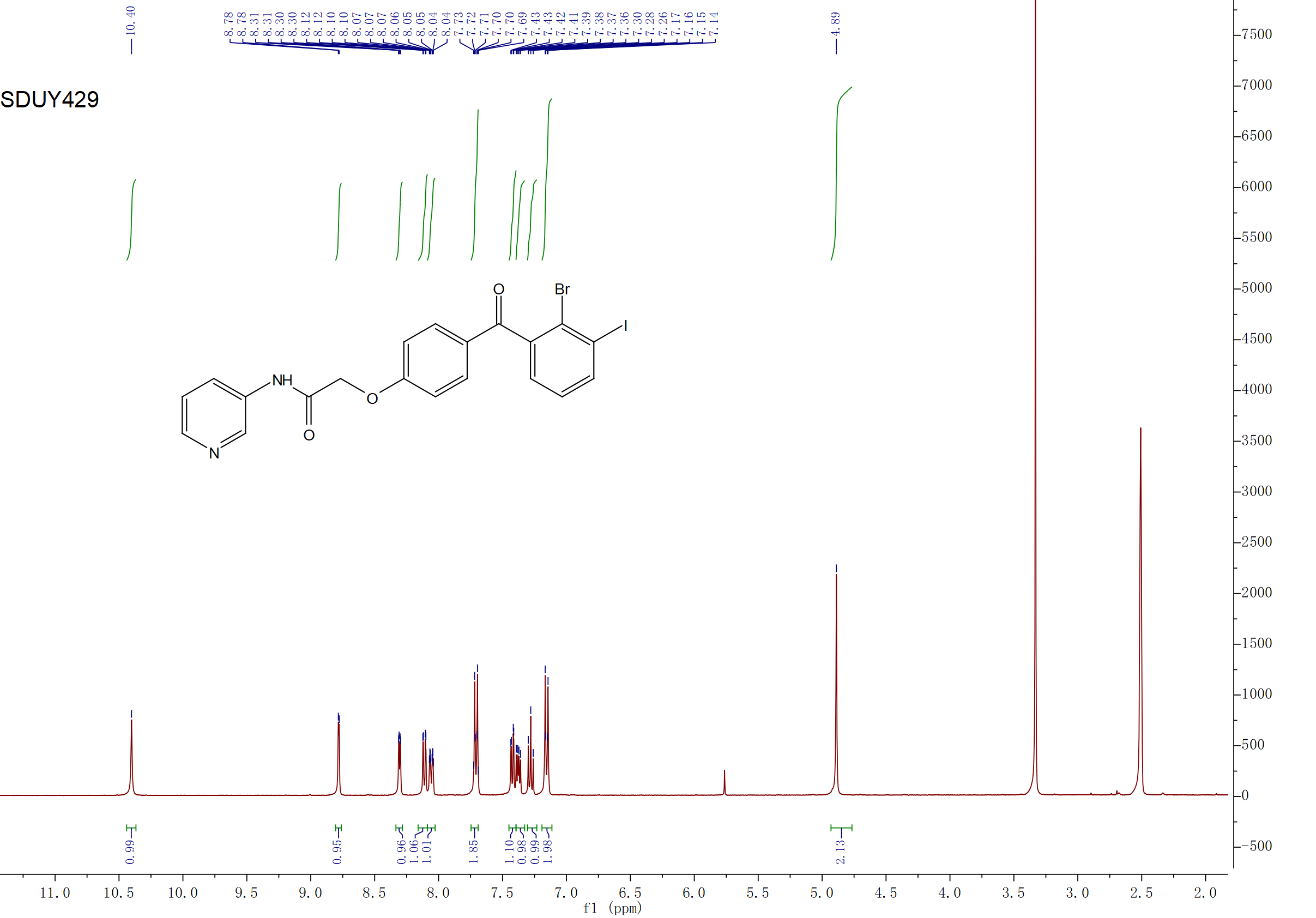


The ^1^H NMR of **SDUY436**


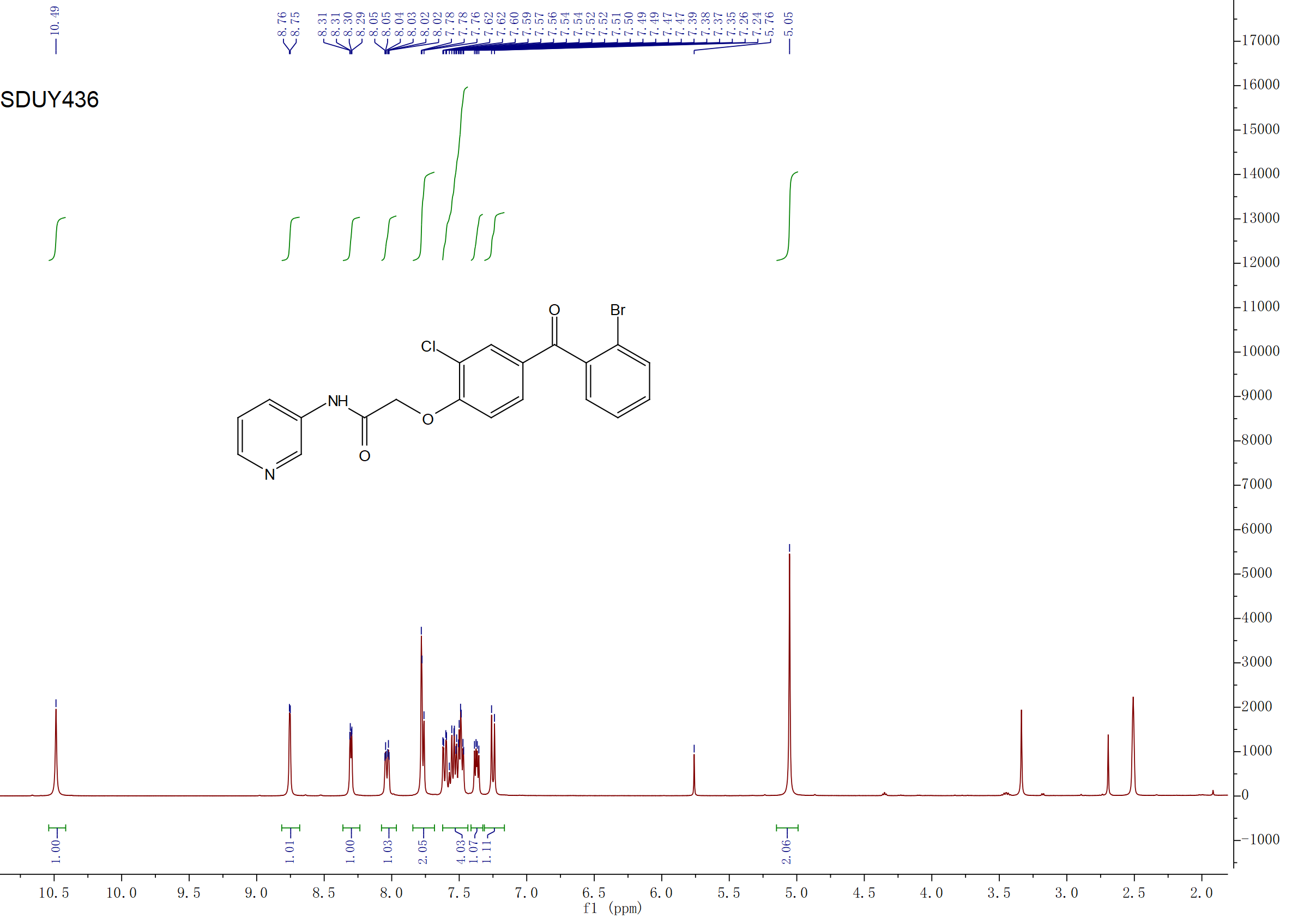


The ^13^C NMR of **SDUY429**


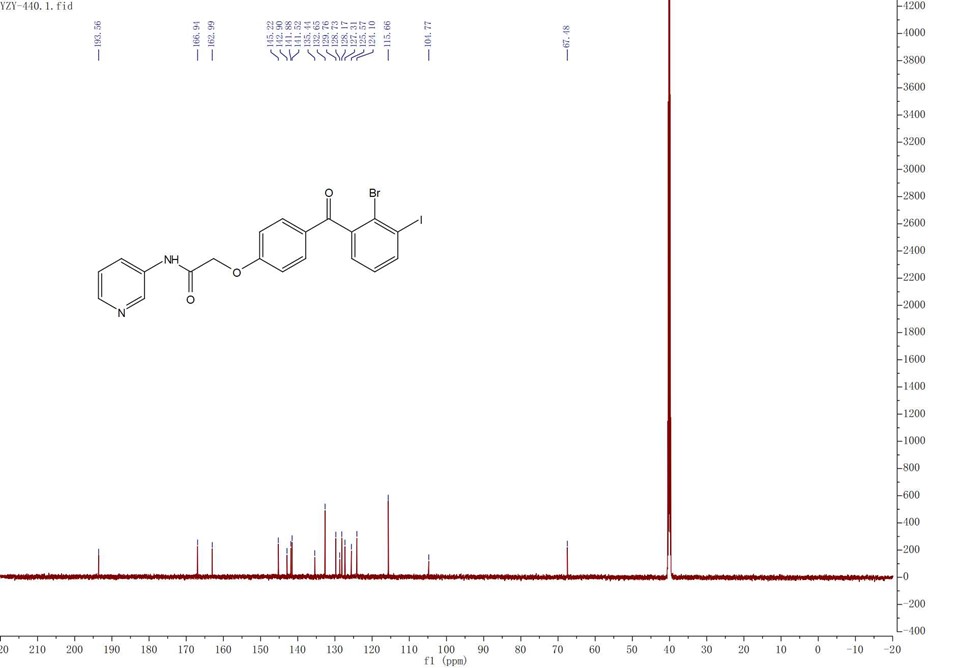


The ^13^C NMR of **SDUY436**


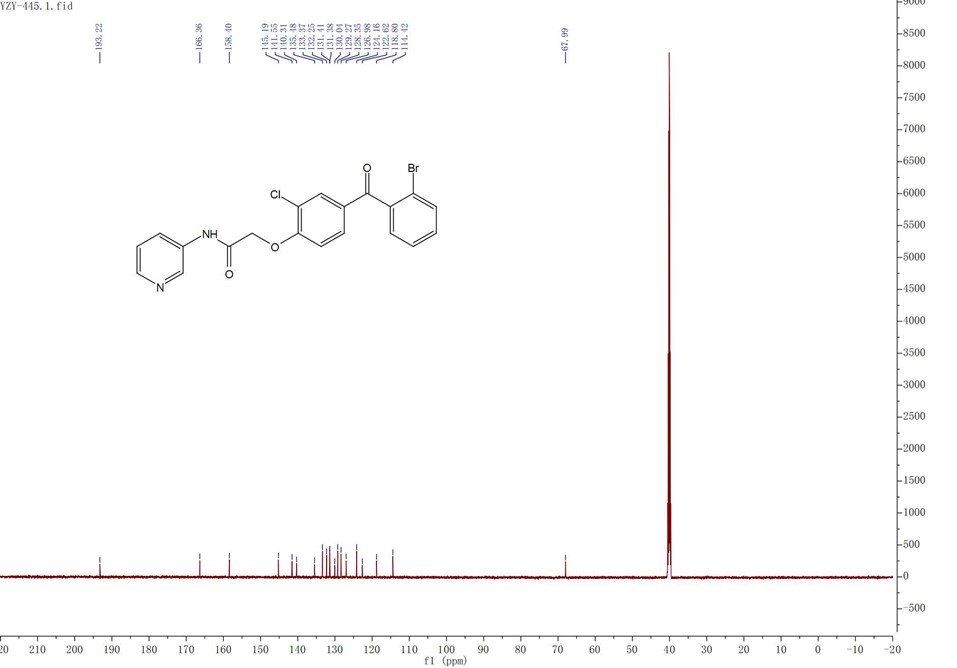

Supplement: Supplementary file 1 [file DataSheet1.doc]
